# A quasi-bridge to surgery approach for stage IV obstructive colon cancer: extending the bridge-to-surgery concept to metastatic disease

**DOI:** 10.1186/s12957-026-04492-3

**Published:** 2026-07-09

**Authors:** Shu-Huan Huang, Sum-Fu Chiang, Kun-Yu Tsai, Pao-Shiu Hsieh, Jeng-Fu You, Cheng-Chou Lai

**Affiliations:** 1 Division of Colon and Rectal Surgery, Department of Surgery, Chang Gung Memorial Hospital, Linkou, and College of Medicine, Chang Gung University, No. 5, Fuxing Street, Guishan District, Taoyuan City, 333 Taiwan; 2 School of Traditional Chinese Medicine, College of Medicine, Chang Gung University, Taoyuan, 33302 Taiwan; 3 Division of Colon and Rectal Surgery, Department of Surgery, New Taipei Municipal Tucheng Hospital, No. 6, Sec. 2, Jincheng Rd., Tucheng Dist, New Taipei City, 236 Taiwan

**Keywords:** Colorectal cancer, Self-expandable metallic stent, Bridge to surgery, Stage IV, Primary tumor resection, Malignant large bowel obstruction

## Abstract

**Background:**

The optimal management of stage IV colorectal cancer with malignant large bowel obstruction remains controversial. Self-expandable metallic stents are an established bridge to surgery in non-metastatic disease, but the role of primary tumor resection after stent placement in stage IV patients is unresolved. We propose and evaluate a “quasi-bridge to surgery” (Quasi-BTS) strategy in which stenting serves as a biological selection tool to identify patients who may benefit from delayed primary tumor resection.

**Methods:**

This single-center retrospective cohort study analyzed consecutive stage IV obstructive colorectal cancer patients who underwent successful self-expandable metallic stent placement between January 2007 and December 2023. Patients were categorized into a Quasi-BTS group (delayed primary tumor resection), a palliative chemotherapy-only group, and palliative care-only group. Primary outcomes were overall survival, stent-related complications, and stoma-free survival.

**Results:**

Fifty-three patients were analyzed (Quasi-BTS, *n* = 16; palliative chemotherapy only, *n* = 22; palliative care only, *n* = 15). The Quasi-BTS group had zero overall stent-related complications versus 46.7% and 36.4% in the palliative care-only and palliative chemotherapy-only groups, respectively (*P* = 0.009); perforation alone was a non-significant trend (0% vs. 26.7% vs. 27.3%, *P* = 0.070). The laparoscopic surgery rate was 82%, the primary anastomosis rate was 92%, and bevacizumab was safely administered in all 6 patients who received it. Overall survival was significantly longer in the Quasi-BTS group than in either palliative group (log-rank all *P* < 0.001). After multivariable Cox adjustment for age, ASA, metastatic site, and targeted-therapy use, the Quasi-BTS group retained a markedly reduced hazard of death (adjusted HR 0.037, 95% CI 0.010–0.135). The mean interval from stent insertion to resection was 21.2 days in the resection-first subgroup.

**Conclusions:**

The Quasi-BTS strategy may extend the bridge-to-surgery concept to metastatic colorectal cancer by repurposing stenting as a bridge for patient selection. Performing primary tumor resection within approximately three months of stent insertion was associated with the absence of stent-related complications in our cohort, appears to permit the subsequent use of anti-angiogenic targeted therapy without observed stent-related complications, and was associated with improved long-term survival.

**Supplementary Information:**

The online version contains supplementary material available at 10.1186/s12957-026-04492-3.

## Background

Colorectal cancer (CRC) is one of the leading causes of cancer-related mortality worldwide [1]. Approximately 20% of patients present with stage IV disease at initial diagnosis, and 8–29% of these patients develop malignant large bowel obstruction (MLBO), a life-threatening surgical emergency requiring immediate intervention [2, 3].

Emergency surgery has traditionally been the standard treatment for obstructive CRC. However, emergency surgery is typically performed under suboptimal physiological conditions, including dehydration, electrolyte imbalance, and nutritional depletion, and often necessitates Hartmann’s procedure with end-colostomy formation [4, 5]. The associated morbidity and mortality are substantially higher than those of elective surgery [4–6], creating a particularly difficult decision when managing stage IV obstructive patients.

Since their introduction in the 1990s, self-expandable metallic stents (SEMS) have fundamentally changed the management of MLBO [7, 8]. In non-metastatic (stage I–III) CRC, the CReST and ESCO trials have demonstrated that SEMS used as a bridge to surgery (BTS) reduces stoma formation while providing comparable short- and long-term oncological outcomes [6, 9–11]. A systematic review and meta-analysis of randomized controlled trials has confirmed that BTS reduces adverse events and stoma rates compared with emergency surgery for left-sided malignant colonic obstruction [6]. The BTS concept in curable CRC is therefore well established.

In the setting of stage IV metastatic CRC, however, the role of SEMS remains ambiguous. The 2020 European Society of Gastrointestinal Endoscopy (ESGE) guidelines recommend SEMS as the preferred first-line treatment for malignant colonic obstruction in the palliative setting [12], but whether to proceed with primary tumor resection (PTR) after stent-mediated decompression is unresolved.

The debate over PTR in stage IV CRC is ongoing. Proponents argue that PTR prevents future tumor-related complications and may improve response to systemic therapy [13, 14]. Opponents cite the JCOG1007 (iPACS) trial, which found no extension of overall survival with PTR in asymptomatic stage IV patients [15]. A combined analysis of the SYNCHRONOUS and CCRe-IV randomized trials similarly confirmed no overall survival benefit of PTR in asymptomatic stage IV CRC (hazard ratio 0.95; 95% confidence interval 0.743–1.215; *P* = 0.685), with a lower proportion of patients receiving chemotherapy in the surgery group [16]. The CAIRO4 trial reported higher 60-day mortality with PTR (11% vs. 3%; *P* = 0.03) without a survival benefit [17]. These trials, however, enrolled asymptomatic patients without acute obstruction — a fundamentally different clinical scenario from the symptomatic obstructive patients addressed in the present study.

Stent-related complication rates rise substantially with prolonged stent dwell time. A meta-analysis by van Halsema et al. demonstrated that stent-related perforation occurs in approximately 7.4% of patients overall, with bevacizumab use and prolonged stent duration identified as independent risk factors [18]. In patients receiving anti-angiogenic agents with retained stents, perforation rates increase to 12.5%, compared with 7.0% in those not receiving bevacizumab [18, 19]. This creates a clinical paradox: current National Comprehensive Cancer Network (NCCN) and European Society for Medical Oncology (ESMO) guidelines strongly recommend first-line bevacizumab for metastatic CRC [20, 21], yet ESGE guidelines caution against bevacizumab use after stent placement owing to the elevated perforation risk [12].

We propose the concept of a “quasi-bridge to surgery” (Quasi-BTS), specifically referring to the use of SEMS in stage IV obstructive CRC as a biological bridge. During a bridging evaluation period of typically 2 to 4 weeks, the stent provides decompression while enabling observation of tumor biological behavior and systemic therapy response. This process functions as a form of biological selection: if the tumor responds favorably and the disease remains stable, delayed PTR is pursued; if the tumor progresses rapidly, surgery is avoided, and the patient remains in the palliative pathway. A critical advantage is that the primary tumor and the stent are removed approximately 3 months after insertion, thereby substantially minimizing stent-related complications [18, 22, 23].

The aim of the present study was to evaluate the clinical efficacy of the Quasi-BTS strategy in stage IV obstructive CRC, comparing survival, stent-related complications, stoma-free survival, and systemic therapy tolerance between treatment groups.

## Methods

### Study design and patient population

This retrospective cohort study was conducted at a single tertiary medical center (Chang Gung Memorial Hospital, Linkou, Taiwan). Between January 2007 and December 2023, all consecutive stage IV obstructive CRC patients who underwent SEMS placement as initial decompression therapy were identified from our prospective colorectal cancer database. The study was approved by the Institutional Review Board of Chang Gung Memorial Hospital (IRB No. 202501451B0) and was conducted in accordance with the Declaration of Helsinki.

Inclusion criteria were: (1) pathologically confirmed adenocarcinoma of the colon or rectum; (2) stage IV disease according to the American Joint Committee on Cancer (AJCC) 8th edition staging system at diagnosis; (3) clinical presentation of MLBO with a Colorectal Obstruction Scoring System (CROSS) score ≤ 1; and (4) successful SEMS placement as initial decompressive therapy.

Exclusion criteria were: (1) perforation or peritonitis at presentation requiring emergency surgery; (2) tumor location less than 5 cm from the anal verge; (3) technical failure of stent placement or lack of clinical improvement within 72 h; and (4) concurrent active non-colorectal malignancy.

### Group definitions

**Fig. 1:**
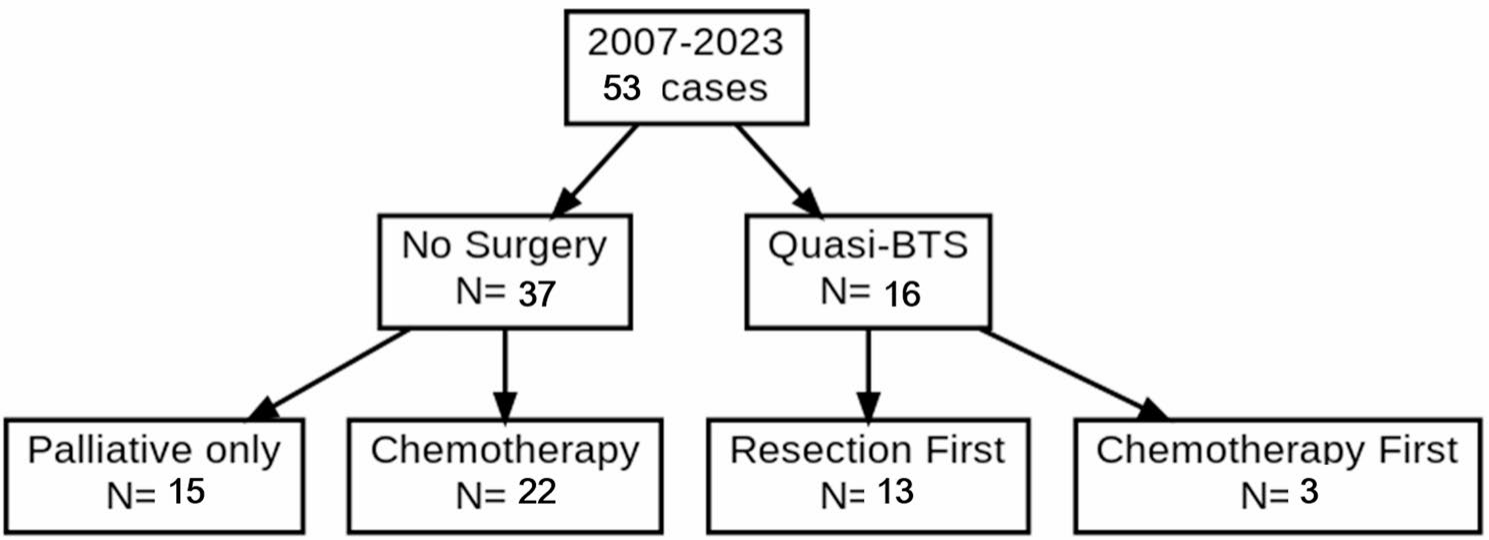
Patient flow diagram. Of 53 stage IV obstructive colorectal cancer patients who underwent successful self-expandable metallic stent (SEMS) placement between 2007 and 2023, 37 did not undergo primary tumor resection (no-surgery group: palliative care only, *n* = 15; palliative chemotherapy only, *n* = 22) and 16 underwent delayed primary tumor resection (Quasi-BTS group: resection first, *n* = 13; chemotherapy first, *n* = 3). BTS, bridge to surgery

On the basis of the clinical pathway followed after SEMS placement, patients were categorized into three groups (Fig. 1).

#### Quasi-BTS group

Patients who, after stent-mediated decompression, underwent a period of systemic evaluation or induction chemotherapy and ultimately received delayed PTR. Within this group, patients were further subdivided into a “resection-first” subgroup (*n* = 13) and a “chemotherapy-first” subgroup (*n* = 3) according to whether surgery or systemic therapy was initiated first after stent placement.

#### Palliative chemotherapy-only group

Patients who did not undergo PTR after stent decompression but received systemic chemotherapy with the stent retained.

#### Palliative care-only group

Patients who received neither PTR nor systemic chemotherapy and were managed with best supportive care alone.

### Endpoints

The primary endpoint was overall survival (OS), defined as the interval from SEMS placement to death or last follow-up. Secondary endpoints were: (1) stoma-free survival; (2) stent-related complications, including perforation, migration, and re-obstruction; (3) time to initiation of systemic chemotherapy and the rate and safety of bevacizumab and anti–epidermal growth factor receptor (EGFR) agent administration; and (4) perioperative surgical outcomes, including laparoscopic surgery rate, intraoperative blood loss, primary anastomosis rate, and mechanical bowel preparation rate.

Additional baseline variables were extracted from the prospective database and medical records, including metastatic disease burden (single- versus multi-organ involvement and the number of involved metastatic organs), hepatic metastatic burden, secondary curative-intent treatment, molecular markers, performance status, and nutritional status. Hepatic metastatic burden was graded according to the Japanese Society for Cancer of the Colon and Rectum (JSCCR) H classification: H1, four or fewer liver metastases with a maximum diameter ≤ 5 cm; H3, five or more metastases with a maximum diameter > 5 cm; and H2, disease not fulfilling the criteria for H1 or H3. Secondary curative-intent treatment comprised secondary metastasectomy or ablation, conversion surgery (in chemotherapy-first patients), and achievement of no evidence of disease (NED), defined as no clinically or radiologically detectable residual disease after treatment, achieved either by complete (R0) surgical resection or ablation of all macroscopic primary and metastatic tumor, or by a complete response to systemic therapy. Molecular markers (RAS, BRAF, and microsatellite-instability status), Eastern Cooperative Oncology Group (ECOG) performance status, and pre-stent serum albumin were recorded where available, and the extent of missing data was reported.

### Statistical analysis

Continuous variables were expressed as means with standard deviations and compared using one-way ANOVA or the Kruskal–Wallis test as appropriate. For the specific comparison of the interval to stent-related perforation between the two palliative subgroups, the Mann–Whitney U test with exact P-value was applied owing to the small subgroup sizes (*n* = 4 and *n* = 6) and heterogeneous variances; values for this variable are presented as median (interquartile range). Categorical variables were expressed as frequencies with percentages and compared using the χ² test or Fisher’s exact test. Survival was analyzed using the Kaplan–Meier method with log-rank comparisons. Multivariable Cox proportional hazards regression was performed to adjust the overall survival comparison for potential confounders, including age (≥ 80 vs. < 80 years), ASA classification (I/II/III), presence of liver, lung, or peritoneal metastases, and use of anti-VEGF or anti-EGFR targeted therapy; the palliative care-only group served as the reference category for treatment group comparisons, and ASA I served as the reference category for ASA classification. Proportional hazards assumptions were verified graphically using log-minus-log survival plots. Statistical significance was set at two-sided *P* < 0.05. A 90-day landmark sensitivity analysis was performed to mitigate immortal time bias inherent to the Quasi-BTS definition. Patients who died within 90 days of stent placement were excluded, and overall survival was recalculated from the landmark time onward. Cox regression was repeated using both the full multivariable model (matching the primary analysis covariates) and a parsimonious model restricted to treatment group, age, liver metastasis, and anti-VEGF/EGFR therapy use, to address residual concerns regarding the events-per-variable ratio.

In addition, an exploratory univariable analysis comparing patients who did versus did not enter the Quasi-BTS pathway was performed using the χ² or Fisher exact test for categorical variables and the Mann–Whitney U test for continuous variables, without adjustment for multiplicity.

All analyses were performed using SPSS version 29.0 (IBM Corp., Armonk, NY, USA).

## Results

### Patient enrolment and baseline characteristics

A total of 53 patients met the inclusion criteria and were included for analysis: Quasi-BTS group (*n* = 16), palliative chemotherapy-only group (*n* = 22), and palliative care-only group (*n* = 15). The patient flow is shown in Fig. 1. Within the Quasi-BTS group, 13 patients underwent resection first, and 3 underwent chemotherapy first, then PTR.

Baseline patient characteristics are summarized in Table 1. Age, sex, tumor location, and pre-stent carcinoembryonic antigen (CEA) levels were comparable across groups (all *P* > 0.05); among metastatic sites, only the proportion with ‘other’ (brain, spleen, adrenal) sites differed significantly across groups, being confined to the palliative care-only group (*P* = 0.005). The mean ages were 70.6 ± 12.5, 69.5 ± 12.6, and 79.2 ± 11.9 years for the Quasi-BTS, palliative chemotherapy-only, and palliative care-only groups, respectively (*P* = 0.059). The sigmoid colon was the most common tumor location across all groups. The liver was the most common metastatic site overall (93.3%, 81.8%, and 62.5% in the palliative care-only, palliative chemotherapy-only, and Quasi-BTS groups; *P* = 0.108). The Quasi-BTS group tended to carry the lowest metastatic burden, with fewer involved metastatic organs (mean 1.31 ± 0.60 vs. 1.93 ± 1.10 and 1.82 ± 0.91; *P* = 0.141), a lower proportion of multi-organ disease (25.0% vs. 46.7% and 54.5%; *P* = 0.192), and a more favorable hepatic burden distribution (H1 grade in 50.0% of patients with liver metastases vs. 28.6% and 16.7%; *P* = 0.195). Baseline performance and nutritional status also favored the Quasi-BTS group, with ECOG 0–1 in 81.2% versus 70.0% and 50.0% (*P* = 0.207) and a higher mean pre-stent serum albumin (3.94 ± 0.46 vs. 3.65 ± 0.51 and 3.18 ± 0.41 g/dL; *P* = 0.003). American Society of Anesthesiologists (ASA) classification differed significantly across groups (*P* = 0.032), with a higher proportion of ASA III patients in both the Quasi-BTS (75.0%) and palliative care-only (73.3%) groups.

**Table 1:** Baseline patient characteristics

Characteristic	Palliative care only (*n* = 15)	Palliative chemotherapy only (*n* = 22)	Quasi-BTS (*n* = 16)	*P* value
Age, years, mean (SD)	79.2 (11.9)	69.5 (12.6)	70.6 (12.5)	0.059
Sex, n (%)				0.275
Male	6 (40.0)	12 (54.5)	11 (68.8)	
Female	9 (60.0)	10 (45.5)	5 (31.3)	
Tumor location, n (%)				0.400
Ascending colon	0	3 (13.6)	0	
Transverse colon	0	2 (9.1)	0	
Descending colon	3 (20.0)	4 (18.2)	4 (25.0)	
Sigmoid colon	10 (66.7)	11 (50.0)	11 (68.8)	
Rectum	2 (13.3)	2 (9.1)	1 (6.3)	
Metastatic site, n (%)				
Liver	14 (93.3)	18 (81.8)	10 (62.5)	0.108
Lung	6 (40)	8 (36.4)	2 (12.5)	0.170
Peritoneum	4 (26.7)	8 (36.4)	4 (25.0)	0.748
Bone	0	1 (4.5)	2 (12.5)	0.308
Distant lymph node	0 (0)	4 (18.2)	2 (12.5)	0.297
Ovary	1 (6.7)	1 (4.5)	1 (6.2)	1.000
Otherᵃ	4 (26.7)	0 (0)	0 (0)	0.005
Multi-organ metastasis, n (%)	7 (46.7)	12 (54.5)	4 (25.0)	0.192
Number of metastatic organs, mean (SD)	1.93 (1.10)	1.82 (0.91)	1.31 (0.60)	0.141
Liver burden(%^*^)				
H1 H2 H3	4 (28.6) 4 (28.6) 6 (42.9)	3 (16.7) 2 (11.1) 13 (72.2)	5 (50) 1 (10) 4 (40)	0.195
ECOG 0–1, n (% of known)^b^	7 (50.0)	14 (70.0)	13 (81.2)	0.207
ASA classification, n (%)				0.032
I	0	4 (18.2)	3 (18.8)	
II	4 (26.7)	8 (36.4)	1 (6.3)	
III	11 (73.3)	10 (45.5)	12 (75.0)	
Pre-stent CEA ≥ 10 ng/mL, n (%)	9 (60.0)	12 (54.5)	9 (56.3)	0.947
Serum albumin, g/dL, mean (SD)^b^	3.18 (0.41)	3.65 (0.51)	3.94 (0.46)	0.003

*ASA* American Society of Anesthesiologists, *BTS* Bridge to surgery, *CEA* Carcinoembryonic antigen, *SD* Standard deviation

^a^Other = brain, spleen, adrenal gland, etc

^b^Albumin available 12/15/13; ECOG 14/20/16 in each group

*Percentages represent the proportion of patients with liver metastases

### Treatment outcomes and stent-related complications

Treatment outcomes and stent-related complications are shown in Table 2. All patients in the palliative chemotherapy-only group and 87.5% of the Quasi-BTS group received systemic chemotherapy. Notably, 56.3% of Quasi-BTS patients received anti-EGFR therapy, compared with 9.1% in the palliative chemotherapy-only group (*P* < 0.001); 37.5% received anti–vascular endothelial growth factor (VEGF) therapy, compared with 31.8% in the palliative chemotherapy-only group (*P* = 0.556).

**Table 2:** Treatment outcomes and stent-related complications

Outcome	Palliative care only (*n* = 15)	Palliative chemotherapy (*n* = 22)	Quasi-BTS (*n* = 16)	*P* value
Received systemic chemotherapy, n (%)	0^a^	22 (100.0)	14 (87.5)	< 0.001
Received anti-EGFR therapy, n (%)	0	2 (9.1)	9 (56.3)	< 0.001
Received anti-VEGF therapy, n (%)	0	7 (31.8)	6 (37.5)	0.556
Stoma formation, n (%)	3 (20.0)	4 (18.2)	2 (12.5)	0.841
Stent-related perforation, n (%)	4 (26.7)	6 (27.3)	0	0.070
Any stent-related complication, n (%)	7 (46.7)	8 (36.4)	0	0.009
Interval to perforation, median (IQR)^b^	29.5 (13.8–52.8)	128.0 (36.3–350.0)	N/A	0.067

BTS, bridge to surgery; EGFR, epidermal growth factor receptor; N/A, not applicable; SD, standard deviation; VEGF, vascular endothelial growth factor

^a^Four patients in the palliative care-only group had received chemotherapy before stent insertion; none continued systemic therapy after stent placement

^b^For the interval to stent-related perforation, comparisons between palliative subgroups were made using the Mann–Whitney U test with exact P-value, given small subgroup sizes (*n* = 4 and *n* = 6) and heterogeneous variances. Values are presented as median (interquartile range)

Stent-related complications differed substantially between groups. Stent-related perforation occurred in 26.7% of palliative care-only patients and 27.3% of palliative chemotherapy-only patients, but in no Quasi-BTS patient; this difference in perforation rates alone represented a numerical trend (*P* = 0.070) that did not reach the conventional threshold of statistical significance. However, the overall rate of any stent-related complication (perforation, migration, or re-obstruction) was 46.7% and 36.4% in the palliative care-only and palliative chemotherapy-only groups, respectively, versus 0% in the Quasi-BTS group (*P* = 0.009), a statistically significant reduction. Among patients who developed stent-related perforation, the interval from stent insertion to perforation tended to be shorter in the palliative care-only group than in the palliative chemotherapy-only group (median 29.5 days [IQR 13.8–52.8], *n* = 4, vs. median 128.0 days [IQR 36.3–350.0], *n* = 6; Mann–Whitney U = 3, exact *P* = 0.067). Although this difference did not reach conventional statistical significance in this small subgroup, the numerical trend is consistent with the cumulative nature of stent-related complication risk associated with prolonged stent dwell time.

Stoma formation rates were 20.0%, 18.2%, and 12.5% in the palliative care-only, palliative chemotherapy-only, and Quasi-BTS groups, respectively (*P* = 0.841).

The mean interval from stent insertion to resection was 21.2 days (range, 5–44 days) for patients in the resection-first subgroup. For the three chemotherapy-first patients, the intervals were 110, 212, and 237 days, respectively.

### Secondary curative-intent treatment and molecular characteristics

Secondary curative-intent treatment was confined to the Quasi-BTS group: 8 of 16 patients (50.0%) underwent metastasectomy or ablation and 7 of 16 (43.8%) achieved no evidence of disease (NED) after the treatment, versus none in either palliative group (both *P* < 0.001); 1 of the 3 chemotherapy-first patients underwent conversion surgery. Molecular testing was incomplete, particularly in earlier years and in palliative-care-only patients: RAS status was available in 13 of 16 Quasi-BTS patients (wild-type 9, mutant 4) versus 12 of 22 and 6 of 15 palliative patients, and RAS testing was more complete in the Quasi-BTS group (missing in 18.8% vs. 45.5% and 60.0%; *P* = 0.066). BRAF was assessable in only 5 of 53 patients (no mutations detected) and microsatellite-instability status in 22 of 53 (all microsatellite-stable; no MSI-high tumors). These data are summarized in Supplementary Table S2.

### Survival analysis

**Fig. 2:**
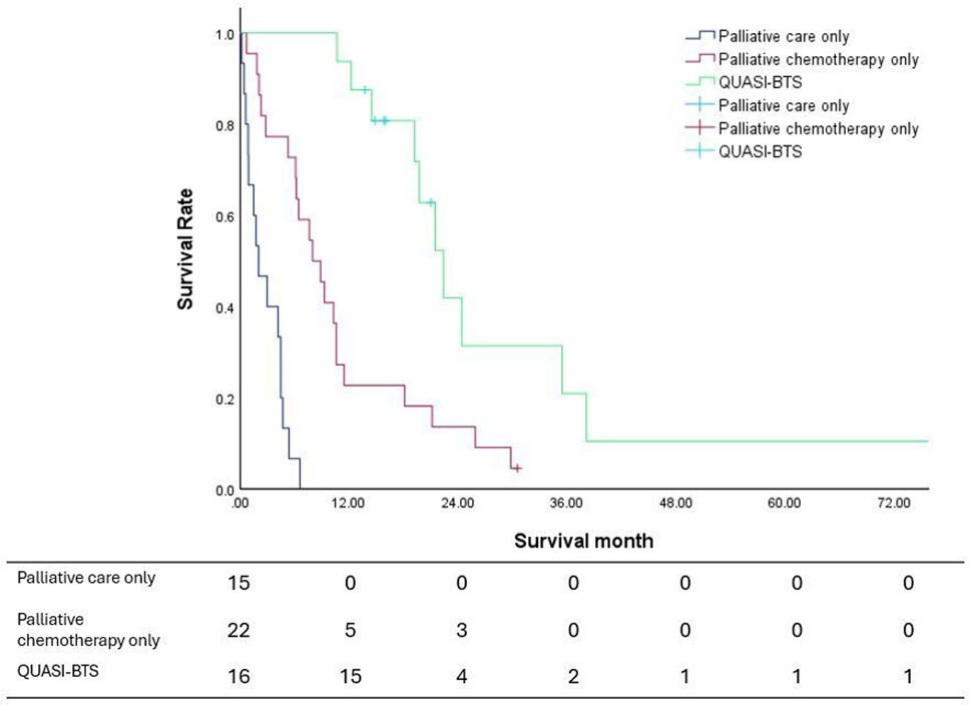
Kaplan–Meier overall survival curves for the Quasi-BTS group (*n* = 16), palliative chemotherapy-only group (*n* = 22), and palliative care-only group (*n* = 15). Overall survival was significantly longer in the Quasi-BTS group than in either palliative group (all *P* < 0.001). BTS, bridge to surgery

Kaplan–Meier analysis demonstrated significantly superior overall survival in the Quasi-BTS group compared with both palliative groups (all *P* < 0.001; Fig. 2). The median overall survival was 22.4 months in the Quasi-BTS group, 8.0 months in the palliative chemotherapy-only group, and 2.0 months in the palliative care-only group.

### Multivariable analysis of overall survival

Multivariable Cox proportional hazards regression was performed to adjust the overall survival comparison for potential confounders (Table 3). After adjustment for age, ASA classification, metastatic site, and use of anti-VEGF or anti-EGFR targeted therapy, the Quasi-BTS group demonstrated a markedly reduced hazard of death compared with the palliative care-only reference group (adjusted HR 0.037, 95% CI 0.010–0.135, *P* < 0.001). The palliative chemotherapy-only group also showed significantly improved survival relative to palliative care-only (adjusted HR 0.152, 95% CI 0.051–0.451, *P* < 0.001). Use of anti-VEGF or anti-EGFR therapy showed a trend toward improved survival that did not reach significance in the full model (adjusted HR 0.46, 95% CI 0.21–1.03, *P* = 0.059). Age ≥ 80 years, ASA classification, and individual metastatic sites (liver, lung, peritoneum) were not independently associated with survival in the adjusted model.

**Table 3:** Multivariable Cox regression analysis of overall survival

	HR	95% CI	*p*-value
Palliative care only	1		
Palliative chemotherapy only	0.152	0.051–0.451	< 0.001
Quasi-BTS	0.037	0.010–0.135	< 0.001
ASA			
I	1		
II	0.951	0.292–3.099	0.934
III	0.831	0.373–1.851	0.650
Use anti-VEGF or anti-EGFR	0.46	0.21–1.03	0.059
Age ≥ 80 years	1.93	0.84–4.48	0.123
Liver metastasis	2.58	0.82–8.15	0.106
Lung metastasis	0.64	0.31–1.32	0.224
Peritoneal metastasis	2.23	0.87–5.73	0.095

*ASA* American Society of Anesthesiologists, *BTS* Bridge to surgery, *CI* Confidence interval, *EGFR* Epidermal growth factor receptor, *HR* Hazard ratio, *VEGF* Vascular endothelial growth factor

#### Sensitivity analyses for immortal time bias

In the 90-day landmark cohort, 14 of 53 patients (26.4%) who died within 90 days of stent placement were excluded; the resulting cohort comprised 39 patients (palliative care-only, *n* = 6; palliative chemotherapy-only, *n* = 17; Quasi-BTS, *n* = 16). All 16 Quasi-BTS patients (100%) reached the 90-day landmark, compared with 6 of 15 (40.0%) palliative care-only and 17 of 22 (77.3%) palliative chemotherapy-only patients, quantifying the magnitude of immortal time embedded in the Quasi-BTS definition. Despite this exclusion, the Kaplan–Meier log-rank test demonstrated a significant difference between groups (χ² = 48.67, df = 2, *p* < 0.001), and multivariable Cox regression of the landmark cohort retained a markedly reduced adjusted hazard of death for the Quasi-BTS group, both in the full model (adjusted HR 0.005, *p* < 0.001) and in a parsimonious model (adjusted HR 0.014, *p* < 0.001) restricted to four covariates to address the limited events-per-variable ratio of the full model, versus palliative care-only. Use of anti-VEGF or anti-EGFR therapy remained significantly associated with survival in the parsimonious landmark model (adjusted HR 0.347, *p* = 0.034). These results support that the observed survival advantage is not driven solely by immortal time bias.

### Factors associated with quasi-BTS selection

On exploratory univariable analysis (Supplementary Table S3), patients selected for the Quasi-BTS pathway had a significantly lower metastatic burden (mean 1.31 vs. 1.86 involved organs; *p* = 0.049) and a higher serum albumin (3.94 vs. 3.44 g/dL; *P* = 0.008), with non-significant trends toward fewer liver (62.5% vs. 86.5%; *p* = 0.068) and lung metastases (12.5% vs. 37.8%; *p* = 0.103) and a lower rate of ECOG ≥ 2 (18.8% vs. 35.1%; *p* = 0.333). The proportion of RAS wild-type tumors did not differ among tested patients (*p* = 0.484). Given the small sample size, these associations are hypothesis-generating and were not entered into a multivariable predictive model.

## Discussion

The present study demonstrates the feasibility and potential advantages of the Quasi-BTS strategy as a treatment approach for stage IV obstructive CRC. This strategy addresses not merely the question of “when to operate” but more fundamentally the question of “how to decide.”

Sekioka et al. [24] characterized SEMS primarily as a temporal bridge, using stent-mediated surgical delay to improve perioperative outcomes. The present study conceptualizes the interval between stent placement and resection as a biological observation period. In stage IV cancer, time provides a unique diagnostic opportunity: emergency surgery precludes assessment of tumor natural history and response to systemic therapy [4, 5]. In the Quasi-BTS cohort, the interval between stent insertion and resection (mean 21.2 days in the resection-first subgroup) coincided with stent-mediated decompression, initiation of induction chemotherapy, and clinical reassessment, during which patients who exhibited a favorable response profile — including declining CEA levels [25], nutritional recovery, and disease stability on imaging — were preferred candidates for delayed resection, whereas those with rapid progression remained in the palliative pathway.

We acknowledge important limitations in our framing of “biological selection.” Selection of patients for delayed resection was based on the physician’s clinical judgment and retrospective assessment rather than on prospective protocol-defined response criteria. We did not pre-specify CEA decline thresholds, imaging response criteria (RECIST), or nutritional parameters for surgical decision-making. Future prospective trials should pre-specify biological endpoints — including CEA kinetics, volumetric response assessment (RECIST), nutritional recovery (serum albumin, body mass index), and ECOG performance status trajectory — to formally test whether biological selection predicts benefit from delayed surgical resection. Until such validation is available, the superior survival observed in the Quasi-BTS group should be interpreted as suggestive rather than conclusive evidence of a biology-guided surgical strategy [15–17].

Both the present study and Sekioka et al. [24] examined SEMS-mediated bridge-to-surgery in stage IV obstructive colorectal cancer using retrospective cohort analysis. However, the Quasi-BTS framework emphasizes three distinct elements. First, the inter-stent interval (mean 21.2 days in the resection-first subgroup) is explicitly framed as a temporal window for biological observation and patient selection, rather than merely as a perioperative optimization interval. Second, the present study foregrounds the safety of anti-angiogenic therapy after resection, reporting successful bevacizumab administration in all six Quasi-BTS patients who received it. This directly addresses a clinical guideline tension: NCCN guidelines recommend bevacizumab-containing regimens for metastatic CRC [20], whereas ESGE guidelines caution against bevacizumab use in stent-bearing patients owing to elevated perforation risk [12]. Our data suggest that timely stent removal through elective surgery safely permits subsequent anti-angiogenic therapy. Third, the Quasi-BTS approach reconceptualizes the self-expanding stent: from a palliative endpoint (comfort-care decompression) to a biological gate (selection for curative-intent surgery). We nevertheless underscore that the biological selection model remains observational and retrospective, and prospective multicenter trials with pre-defined biological criteria are essential before the Quasi-BTS approach can be recommended as standard practice.

To contextualize the surgical safety of the Quasi-BTS strategy, perioperative outcomes were compared with the emergency-surgery cohort reported by Sekioka et al. [24]. The Quasi-BTS group demonstrated markedly superior perioperative metrics: a laparoscopic surgery rate of 82% versus 19%, intraoperative blood loss of 55 mL versus 292 mL, a primary anastomosis rate of 92% versus approximately 60%, and a mechanical bowel preparation rate of 95% versus 19%. These findings support the view that, even in stage IV patients, stent-mediated decompression converts what would otherwise be an emergency operation into an elective, minimally invasive procedure, preserving the benefits of enhanced recovery protocols [4, 6, 10]. The ability to achieve primary anastomosis in the vast majority of Quasi-BTS patients is particularly important, because it directly contributes to improved stoma-free survival and quality of life.

The higher utilization of anti-EGFR therapy in the Quasi-BTS group (56.3% vs. 9.1% in the palliative chemotherapy-only group; *P* < 0.001) likely reflects both molecular selection (patients with RAS wild-type, left-sided tumors who are optimal candidates for anti-EGFR agents were more often deemed suitable for curative-intent delayed resection) and survival-dependent treatment exposure (longer survival provided greater opportunity for second- or third-line anti-EGFR regimens). The extent to which this difference mediates the observed survival advantage requires further investigation in larger, molecularly annotated cohorts.

Part of the survival advantage observed in the Quasi-BTS group is likely mediated by greater access to curative-intent secondary treatment: half of these patients underwent metastasectomy and 43.8% achieved NED, a state associated with markedly superior survival in metastatic colorectal cancer. This is a downstream consequence of — rather than an alternative to — favorable biological selection, and the survival differences should therefore not be attributed to delayed primary tumor resection alone.

Consistent with this interpretation, exploratory analyses indicated that the pathway preferentially captured patients with lower-burden disease, better performance status and better nutritional status. While this is the explicit intent of a biological-selection strategy, it also limits the generalizability of our findings to patients with higher metastatic burden or poorer performance status, in whom the risk–benefit balance of delayed resection may differ.

The Quasi-BTS approach may offer a practical approach to a challenging clinical dilemma. Current NCCN guidelines (version 3.2024) recommend first-line bevacizumab-containing regimens for most patients with metastatic CRC [20], while ESGE guidelines simultaneously caution against bevacizumab use after stent placement because of an elevated perforation risk [12]. A meta-analysis has confirmed that the stent-related perforation risk increases substantially with anti-VEGF therapy, with rates as high as 12.5% in bevacizumab-treated patients with retained stents [18]. By removing the primary tumor and the stent through Quasi-BTS, this interaction triangle may be interrupted, potentially creating a more favorable physiological environment for subsequent systemic therapy. This perspective challenges conclusions drawn from JCOG1007 [15] and the SYNCHRONOUS/CCRe-IV trials [16], which did not consider the specific clinical scenario of obstructive patients with retained stents or the interaction between stents and targeted agents.

Our data support the concept that performing surgery within approximately three months of stent insertion represents a critical threshold. Long-term stent complication data consistently show that perforation, migration, and tumor ingrowth increase progressively with dwell time [18, 22, 23]. In our palliative groups, the interval to stent-related perforation was numerically shorter in the palliative care-only group (median 29.5 days) than in the palliative chemotherapy-only group (median 128.0 days; exact *P* = 0.067), a trend consistent with — although not conclusive of — the hypothesis that chemotherapy may partially control local tumor progression without preventing the mechanical complications inherent to prolonged stent presence. The complete absence of stent-related complications in the Quasi-BTS group (0% vs. 36.4–46.7%; *P* = 0.009) is consistent with the hypothesis that timely stent removal through planned surgery may reduce stent-related complications, although confirmation in larger prospective cohorts is required.

Although the palliative groups initially avoided surgery, modern systemic therapy extends median survival beyond 30 months in metastatic CRC [20, 21]. As survival lengthens, the cumulative risk of stent failure becomes substantial [7, 22] and often necessitates emergency salvage stoma creation under compromised conditions. By contrast, the Quasi-BTS group, through elective minimally invasive surgery with primary anastomosis (92%), largely avoided stoma formation. For stage IV patients with relatively longer expected survival, Quasi-BTS therefore represents an attractive strategy for preserving long-term quality of life [10, 26].

The survival advantage of the Quasi-BTS group persisted after multivariable adjustment for ASA classification and use of targeted therapy (Table 3), with a markedly reduced adjusted hazard of death (HR 0.037, 95% CI 0.010–0.135, *P* < 0.001) relative to the palliative care-only group. Palliative chemotherapy alone also conferred an independent survival benefit (HR 0.152, 95% CI 0.051–0.451, *P* < 0.001), consistent with the established benefit of modern systemic therapy in metastatic CRC [20, 21]. These adjusted estimates support the robustness of the primary survival finding against the baseline imbalances observed between groups.

The 90-day landmark sensitivity analysis directly addresses concerns regarding immortal time bias. Whereas only 40.0% of palliative care-only patients survived to the 90-day landmark, all Quasi-BTS patients did so by definition, quantifying the immortal time embedded in any strategy requiring survival until planned delayed resection. Crucially, after structurally removing this bias by excluding all early deaths, the Quasi-BTS group retained an extreme adjusted survival benefit (full-model HR 0.005, parsimonious HR 0.014; both *P* < 0.001 versus palliative care-only), with a concordant log-rank *P* < 0.001. The concordance between the full and parsimonious models — the latter with a more favorable events-per-variable ratio — argues that the magnitude of the effect estimate is not an artefact of overfitting. Together with the multivariable adjustment for measured confounders in the primary analysis, these findings provide reassurance that the survival advantage of the Quasi-BTS strategy reflects the impact of the strategy itself rather than purely structural selection.

Several limitations should be acknowledged. First, this is a retrospective, single-center study with a relatively small sample size and is inherently subject to selection bias. The superior outcomes in the Quasi-BTS group may partly reflect physician selection of patients with a more favorable prognosis — although this is in fact the core logic of the strategy. Second, with 53 patients and multiple covariates in the Cox model, the events-per-variable ratio is suboptimal; the adjusted hazard ratios should therefore be interpreted with caution and confirmed in larger cohorts. Third, our framing of “biological selection” is post-hoc and observational: no prospective criteria (CEA kinetics, RECIST response, nutritional or performance-status thresholds) were pre-specified for surgical decision-making. Fourth, the Quasi-BTS group is inherently subject to immortal time bias, because patients by definition had to survive long enough to undergo the planned delayed primary tumor resection (mean interval 21.2 days). Deaths occurring in this interval would have been classified in the palliative pathway. Although a 90-day landmark sensitivity analysis was performed (see Supplementary Material) and supported the primary finding, readers should interpret the between-group survival comparisons with this structural bias in mind. Fifth, the study period spans 16 years, during which systemic therapy regimens have evolved substantially. Prospective multicenter studies or randomized trials with pre-defined biological endpoints are needed to validate the Quasi-BTS approach, particularly across different molecular subtypes (MSI-H, BRAF-mutant, RAS-mutant tumors). Sixth, molecular profiling was incomplete: RAS, BRAF and microsatellite-instability testing was not performed routinely during the earlier years of the study period, and patients triaged to palliative care alone were frequently not tested; BRAF was assessable in only 5 of 53 patients (9.4%) and microsatellite-instability status in only 22 of 53 (41.5%). The resulting high proportion of missing molecular data, particularly for BRAF and MSI, limits interpretation of the observed differences in molecular status and of the higher anti-EGFR utilization in the Quasi-BTS group.

## Conclusions

In the management of stage IV obstructive CRC, the Quasi-BTS strategy represents a conceptual shift from anatomical decompression to biology-guided therapy. Our data suggest that in appropriately selected stage IV patients, delayed PTR following stent-mediated decompression was associated with improved survival, no observed stent-related complications, and the safe use of anti-angiogenic targeted therapy. Performing surgery within approximately three months of stent insertion may minimize stent-related morbidity. SEMS should not be viewed merely as the endpoint of palliative treatment but as the starting point of precision medical decision-making.

## Supplementary Information

Supplementary Material 1.

Supplementary Material 2.

Supplementary Material 3.

## Data Availability

No datasets were generated or analysed during the current study.

## Abbreviations

AJCC: American Joint Committee on Cancer
ASA: American Society of Anesthesiologists
BTS: Bridge to surgery
CEA: Carcinoembryonic antigen
CI: Confidence interval
CRC: Colorectal cancer
CROSS: Colorectal Obstruction Scoring System
ECOG: Eastern Cooperative Oncology Group
EGFR: Epidermal growth factor receptor
ESGE: European Society of Gastrointestinal Endoscopy
ESMO: European Society for Medical Oncology
HR: Hazard ratio
MLBO: Malignant large bowel obstruction
NCCN: National Comprehensive Cancer Network
OS: Overall survival
PTR: Primary tumor resection
Quasi-BTS: Quasi-bridge to surgery
SEMS: Self-expandable metallic stent
SFS: Stoma-free survival
VEGF: Vascular endothelial growth factor
