# Supplementary material for: A quasi-bridge to surgery approach for stage IV obstructive colon cancer: extending the bridge-to-surgery concept to metastatic disease
Source: World J Surg Oncol. 2026 Jul 9;24:315. doi: 10.1186/s12957-026-04492-3 (PMC13422036; doi:10.1186/s12957-026-04492-3)
Supplement: Supplementary file 1 — Supplementary Material 1. [file 12957_2026_4492_MOESM1_ESM.docx]

**Supplementary Table S1.** Sensitivity analyses for the 90-day landmark cohort

| **Variable** | **Full model^a^ HR (95% CI), P** | **Parsimonious model^b^ HR (95% CI), P** |
| --- | --- | --- |
| Palliative care-only (reference) | 1.000 | 1.000 |
| Palliative chemotherapy-only | 0.021 (0.003–0.142), <0.001 | 0.051 (0.012–0.227), <0.001 |
| **Quasi-BTS** | 0.005 (0.001–0.044), <0.001 | 0.014 (0.003–0.073), <0.001 |
| Age ≥ 80 years | 7.478 (2.306–24.252), 0.001 | — |
| Age (continuous, years) | — | 1.015 (0.973–1.059), 0.484 |
| ASA II (vs I) | 1.259 (0.304–5.217), 0.751 | — |
| ASA III (vs I) | 0.620 (0.217–1.769), 0.371 | — |
| Liver metastasis | 2.740 (0.624–12.020), 0.182 | 0.952 (0.389–2.331), 0.915 |
| Lung metastasis | 0.484 (0.180–1.299), 0.150 | — |
| Peritoneal metastasis | 2.105 (0.536–8.267), 0.286 | — |
| Use of anti-VEGF or anti-EGFR therapy | 0.535 (0.198–1.447), 0.218 | 0.347 (0.131–0.921), 0.034 |

Cohort: n = 39 (palliative care-only, n = 6; palliative chemotherapy-only, n = 17; Quasi-BTS, n = 16) after exclusion of 14 patients (26.4%) who died within 90 days of stent placement. Reference categories: palliative care-only for treatment group; ASA I for ASA classification.

Kaplan-Meier log-rank: χ² = 48.67, df = 2, P < 0.001.

*a Full model: same covariates as the primary analysis (Table 3).*

*b Parsimonious model: restricted to four key covariates (treatment group, age, liver metastasis, and anti-VEGF/anti-EGFR therapy use) to address the suboptimal events-per-variable ratio in the full model.*

*CI, confidence interval; HR, hazard ratio; VEGF, vascular endothelial growth factor; EGFR, epidermal growth factor receptor; ASA, American Society of Anesthesiologists.*
