# Supplementary material for: A quasi-bridge to surgery approach for stage IV obstructive colon cancer: extending the bridge-to-surgery concept to metastatic disease
Source: World J Surg Oncol. 2026 Jul 9;24:315. doi: 10.1186/s12957-026-04492-3 (PMC13422036; doi:10.1186/s12957-026-04492-3)
Supplement: Supplementary file 2 — Supplementary Material 2. [file 12957_2026_4492_MOESM2_ESM.docx]

# **Supplementary Table S2**. Baseline additions

| **Variable** | **Palliative care only** | **Pal. chemo** | **Quasi-BTS** | **P** |
| --- | --- | --- | --- | --- |
| **Case number** | **15** | **22** | **16** |  |
| **Secondary curative-intent treatment** |  |  |  |  |
| Secondary metastasectomy/ablation, n (%) | 0 (0) | 0 (0) | 8 (50.0) | <0.001 |
| Conversion surgery (chemo-first, n=3) | — | — | 1/3 (33.3) | — |
| NED achieved, n (%) | 0 (0) | 0 (0) | 7 (43.8) | <0.001 |
| **Molecular markers (n)** |  |  |  |  |
| RAS WT/mut/unknown | 1/5/9 | 9/3/10 | 9/4/3 | 0.066ᵃ |
| BRAF WT/mut/unknown | 0/0/15 | 1/0/21 | 4/0/12 | n/a |
| MSI MSS/MSI-H/unknown | 5/0/10 | 4/0/18 | 13/0/3 | n/a |

ᵃ P for difference in proportion of missing RAS data. Fisher exact / Kruskal–Wallis
