# Supplementary material for: A quasi-bridge to surgery approach for stage IV obstructive colon cancer: extending the bridge-to-surgery concept to metastatic disease
Source: World J Surg Oncol. 2026 Jul 9;24:315. doi: 10.1186/s12957-026-04492-3 (PMC13422036; doi:10.1186/s12957-026-04492-3)
Supplement: Supplementary file 3 — Supplementary Material 3. [file 12957_2026_4492_MOESM3_ESM.docx]

# Supplementary Table S3. Factors associated with Quasi-BTS selection

| **Variable** | **Quasi-BTS (16)** | **Non-Quasi-BTS (37)** | **P** |
| --- | --- | --- | --- |
| Multi-organ metastasis, n (%) | 4 (25.0) | 19 (51.4) | 0.130 |
| **No. of metastatic organs, mean (SD)** | **1.31 (0.60)** | **1.86 (0.98)** | **0.049** |
| Liver metastasis, n (%) | 10 (62.5) | 32 (86.5) | 0.068 |
| Lung metastasis, n (%) | 2 (12.5) | 14 (37.8) | 0.103 |
| Peritoneal metastasis, n (%) | 4 (25.0) | 12 (32.4) | 0.748 |
| ECOG ≥ 2, n (%) | 3 (18.8) | 13 (35.1) | 0.333 |
| **Serum albumin, g/dL, mean (SD)** | **3.94 (0.46)** | **3.44 (0.52)** | **0.008** |
| RAS wild-type (of tested) | 9/13 (69.2) | 10/18 (55.6) | 0.484 |
